# On the Study of Nosology, and the Present State of Medical Science

**Published:** 1817-12

**Authors:** 


JOURNAL AND REVIEW.

VOL. IV.] DECEMBER, 1817. [NO. 24.

### PART I.

### ORIGINAL COMMUNICATIONS.

——— quid utile ———

### *On the Study of Nosology, and the present State of Medical Science.* By a PHYSICIAN.*

Αἱ ἀρχαι των αποδειξεων οἱ ὁρισμοι. ARIST.

Ex hujus studii adeo imperfecti conspectu, ni fallor, accidit, quod totam rem Nosologicam alii despexerint, alii vero impossibilem judicaverint. Qui impossibilem putant, certe falluntur.

CULLEN, Syn. N. M. Prolegom.

THE importance of the Study of Nosology has been very differently estimated; and, in too many instances, in my opinion, has been greatly undervalued. It is, therefore, to be hoped, that the learned and elaborate work of Mr. Good will excite a greater attention to, and lead to a higher appreciation of, this branch of medical literature.

Dr. Parr says, "Nosology is seldom mentioned but to be ridiculed and despised. Some late authors, who have pretended to oppose it, have in their arguments shown not only their ignorance, but the necessity of the study."

* The Author is equally well known and respected by the Editors, and this is a sufficient reason for placing the Contribution in the "*Original Department.*" EDITORS.

Without going this length, I must entirely concur in the conclusion, and with those writers who have maintained the advantages which are to be derived from an arrangement sufficiently scientific, and, at the same time, extensive, to merit the appellation of a General System of Diseases.

The errors and imperfections of preceding systems may have shown the infancy of the science, but they are no proof of its inutility; nor do they afford any argument against the extent to which its difficulties may be overcome by unwearied diligence, and gradual approaches.

It has been well remarked, that every thinking practitioner must have formed some kind of nosological system in his own mind—that is, he must have ascertained the symptoms which are characteristic of the different forms of diseases, and which distinguish each from those which resemble it: Nam, si quis hoc negaverit (says Dr. Cullen) idem fecerit, ac si nullam artem medicam dixisset. Si revera tamen medici morbos a se invicem dignoscere possint, certe quibus indiciis id faciant, etiam dicere queunt. Hæc autem indicia nulla alia esse possunt, quam quæ morbum quemque per genus et speciem definiant, quæ rursus per methodum nosologicam rite institutam, solummodo exponi queant. *Syn. Nos. Method. Prolegomina.* xv. The objections that have been raised against Nosology appear to have originated, in a great measure, from limited and imperfect views of its object and powers; by which its true value cannot fail to be depreciated.

It is (to borrow the sentiments of Lord Bacon) from partial and desultory researches that scepticism arises; not only as such researches suggest doubts which a more enlarged acquaintance with the subject would dispel, but as they withdraw the attention from those comprehensive views which combine into a symetrical fabric, all whose parts mutually lend to each other support and stability; the most remote, and seemingly the most unconnected discoveries. "Etenim symetria scientiæ, singulis scilicet partibus se in viam sustinentibus, est, et esse debet, vera atque expedita ratio refellendi objectiones minorum gentium." *De Augment. Scient. Lib.* 1.

It is generally granted, that the object of Nosology is to enable us to arrange, and to discriminate diseases from each other; and, although "the various ills that flesh is heir to," diversified by habit, season, situation, and other modifying circumstances, do not possess that unvarying uniformity of character and appearances which serve to distinguish other branches of knowledge, still these are sufficiently regular and distinctive, to admit of an intelligible classification ; and to enable the science of diseases to be prosecuted with a very beneficial influence upon the practice of medicine. It would, indeed, at first sight, appear paradoxical, that a study, of which the professed object is to direct us in diagnosis, and in practice, should either admit of disparagement, or require encomium ; particularly since its advantages have been long acknowleged by the most eminent physicians at various periods. Without further tracing the causes of this depreciation, which would lead into a wide field, I am inclined to think, that independently of theoretical reasoning, or declamation, on either side, a practical test may be applied, which to me, I confess, appears perfectly conclusive as to the merits of Nosology.

From causes, which it is unnecessary here to specify, I have had extensive opportunities of being acquainted with the professional acquirements of young men entering upon the practice of physic, and of afterwards observing their knowledge put to use at the bed side; and I have generally remarked their judgement in discriminating disease to be clear, and their practice to be judicious, in proportion to their acquaintance with Nosology. It is evident indeed, that a good nosologist at the sick-bed must, cæteris paribus, possess great advantages over one who has neglected this branch of study ; and that the mere recollection of a definition (by embodying the more prominent essential symptoms) will frequently enable him to recognize a disease at once, which, without such assistance, he would have been in danger of either confounding with, or mistaking for, another. By fostering a habit of discrimination, it will also lead him, as Dr. Cullen has adverted to, by investigating, to prescribe for the actual varieties of disease, rather than to content himself with learning their titles, and with considering that every essential information has been acquired, when the name of a disease has been made out ; a habit into which those practitioners, whose education has been conducted without an attention to nosological accuracy, are too liable to fall. Even as aids to the memory then, (as is well observed by the London Medical Review for August 1808) and as simple and comprehensive modes of recalling, and expressing our knowledge, nosological arrangements, when they follow the advances of medicine, must be highly useful.

Such are the uses and tendency of Nosology, when rightly understood ; but I am far from pretending, in the present imperfect state of our knowledge, that it is an infallible guide; or that, (such is often the obscurity, simulation, or complication of disease) it will always enable us either to distinguish a complaint, or help us to the best mode of treatment. I am well aware, that as the human physiognomy is altered by time, by suffering, or by crime, till the picture no longer resembles its original; so, the physiognomy of disease is often altered in its progress by changes and new associations, until it can no longer be recognized by any definition, however faithfully taken; and, therefore, that a too strict adherence to an original delineation may, sometimes, only lead us astray. I am also aware of the errors into which we may fall by incautious generalization; by outstepping, instead of following nature; and of the danger, about which so much has been said, of prescribing for a name—still, "Ex abusu non arguiter ad usum"—and I cannot help thinking, that such objections as have been founded on the abuse of Nosology, cannot afford any legitimate argument against the science itself. That errors, and even imperfections, which may lead us astray, should be avoided; that every system of Nosology contains such errors and imperfections; and, therefore, that the study of Nosology, which may lead us astray, should be avoided —may be syllogistic, but is not inductive logic. It is, to borrow a scholastic axiom, an instance of the "Dictum de omni, et de nullo"—consequently merged in the universality of its application: for, unfortunately, similar objections may be urged, not only against other parts of a science, which, with a mixture of truth and sarcasm, has often been styled "conjectural;" but I fear, it might retort the charge against many branches of the other Sciences, and even of the Arts!

On turning to the Cyclopœdia of Dr. Rees, I was much pleased to find that these remarks are so nearly in accordance with the sentiments therein expressed, upon this subject: "It has been the fashion among many professional men to deny the study of Nosology, and the method of teaching medicine upon a nosological plan; but this can have arisen only from an imperfect acquaintance with its nature and tendency, and a consequent inadequate estimate of its utility. It is necessary, as we have observed above, that every practitioner should be enabled to distinguish diseases by their signs or symptoms, if he would hope to cure them by appropriate remedies. He must therefore possess, in his own mind, an arrangement of the characteristic symptoms of every disease, and especially of the diagnostic symptoms, or those by which it is distinguished from other diseases which resemble it, or with which it has several symptoms in common. The question, therefore, appears to be, whether it is adviseable to teach medicine according to a system in which the diagnostic symptoms are distinctly laid down, or to leave them to be learned by a vague, unmethodical course of reading, and practice? a question which seems to answer itself. We are persuaded, that the great superiority which is commonly seen in practitioners of good education is to be ascribed, in a considerable degree, to the nosological method of studying the principles of medical science, in which they have been early initiated."

The same writer, after adverting to some of the errors into which they, who do not possess this advantage, are liable to be betrayed, judiciously adds: "against such important practical errors the mind is most effectually secured, when it is stored with correct nosological views; for, where these are absent, the practitioner is apt to proceed upon a mere empirical association of certain remedies, with certain prominent symptoms, without duly marking their combinations, or weighing their import; that is, without forming any clear opinion as to the actual seat and nature of the disease."

Among the evils connected with Nosology, and to which a vitiated nomenclature has been supposed to have contributed, the term typhus has been frequently commented upon, as conveying an erroneous idea of the nature, and leading to the improper treatment of the disease which it represents. How such objections are fairly attributable to Nosology, I confess I do not readily perceive; nor, though I am willing to allow due weight to all that has been said of the danger of prescribing for a name, how that has led to the erroneous treatment of the malady in question. We must have names to designate diseases—"Nomina si pereunt, perit etiam cognitio rerum"—but if any other can be shown to be preferable, it would doubtless be adviseable to substitute it for that now in use. If we turn to the etymology of typhus, we find the word to be derived from τυφος, stupor, a degree of sensorial affection which generally supervenes, sooner or later, in most continued fevers; and which is allowed to be a prominent symptom of that to which this name has been especially applied; and were I required to depict this disease in its best marked form, I do not know that it could be done better or in fewer words than in those of Galen, "Affectus ex phrenitide et lethargo mixtus." I do not, therefore, perceive any thing so objectionable in the use of this term, and think that, negatively at least, it might claim the indulgence of being retained, like many others, for want of a better. But we might even go farther, and allow it to have positive merit, if, according to the etymology of Dr. Parr, it were admitted to be derived from τυφω, to inflame; for that typhus is very generally complicated with inflammation in some organ, or with increased determination, or congestion in some part or another, approaching in effect to inflammation, the late researches in pathological anatomy have unquestionably proved; and even where dissection has not discovered such traces of inequality of circulation, it may be inferred to have existed, in a greater or less degree, from analogy, and the phænomena of fever. I do not mean by this remark to identify fever with inflammation, either substantially or in effect, but the admission even of such a concurrence being at least extremely frequent, does not necessarily sanction any inference of the same mode of treatment in all cases being either indispensable or admissible, or that it will be followed by the same results in different epidemics: but a further consideration of this question here would lead from the subject.

The grand error, and which appears to me chiefly to have given rise to the objections against the term typhus, consists in mistaking names for things. Thus with the word typhus has been catenated the notion of debility; but this, it appears, is not sanctioned by its etymology—whence then comes the idea that debility is an essential and characteristic feature of this fever? not surely from the word employed to designate the disease; but from the erroneous theories which have been taught respecting the nature of that disease; and as long as the theories of debility, putrescency, &c. prevailed in the schools of medicine, it is evident, that the treatment must have been in consonance with them, by whatever name the disease might have been known. Such being the supposed nature of fever, to obviate this debility became consequently the curative indication; and the treatment as naturally grew out of the pathology, as in any other instance of the concatenation of cause and effect.

It is from false theories then, and a limited acquaintance with pathological physiology, that incorrect therapeutic views have arisen; from such premises, we cannot be suprized that erroneous conclusions should have flowed, when we recollect the various hypotheses which from the fountain heads of acrimony, lentor, fermentation, putrefaction, &c. winded through all the mazes of the humoral pathology, until they ended in the full stream of Brunonian debility.

It has been proposed to substitute camp, jail, and hospital fever, for typhus. Such epithets convey no determinate idea, and perhaps the most that can be said in their favour is, that they do not pre-suppose any thing as to the nature of the disease; which is a very negative merit indeed. But, although they do not necessarily convey such a meaning, any more than the word typhus does, I might contend, that they are open to the very same objection, that of suggesting, from pre-conceived theory, an idea of debility; for there are, perhaps, few who will be inclined to question that such an idea would not be as likely, and some may think with me, that it would be more likely to recur to the mind, on hearing of a jail, than of a typhus fever: and now, when we are fast disencumbering ourselves of erroneous hypotheses, it would be difficult to shew what advantage would be gained by such a change; particularly since the adjectives, simple, inflammatory, and congestive, so judiciously conjoined with typhus by Dr. Armstrong, and his just and luminous exposition of these states of fever, have more forcibly shown those errors, which are now fading fast away before the light of a better pathology.

When we contemplate the theories which have been taught, implicitly believed, and zealously defended, but which have now, irrevocably, passed away; we turn from them with a mortifying conviction of the proneness to error, and a painful distrust of the stability of speculative opinions; but we dwell upon the revolutions of practical medicine, with which these are connected, as furnishing reflections of greater interest and permanency.

It is said to be a common idea in most countries, which receives countenance from the illustrious author of the inductive philosophy himself, (Bacon's Essays, Art. 59.) and which, from late experience we may feel less inclined to dispute, that after a certain number of years, the same weather and particular seasons (and here the opinions of Sydenham also rise before us) recur, in the same series as before; and when we look back upon the revolutions of our own science, we can hardly refuse assent to Hufeland's kindred idea of a cycle, through which, he imagines, medical opinion has always revolved, but with increasing velocity. Vide Edin. Med. and Surg. Journal, No. 51, for an interesting summary of the revolutions in the treatment of fever.

Without dwelling on the many changes of opinion relative to the utility of blood-letting, and purgatives, in different ages; when we reflect (to mention but a few instances) how nearly the practice of our own time, in fever, approaches to that of the antients, in many respects; that Aræteus recommended washing the body with cold water, or with water and vinegar, in slow nervous fever; that Celsus used the lancet in many of those cases in which it is allowed to be most proper at present; that Botallus brought venæsection into great vogue, when it was nearly altogether superseded by purging; and that he employed it in many diseases to which other bolder practitioners have also, recently extended it: and, finally, that Riverius directed it in the same way, and in cases precisely similar to those in which it is prescribed at present, and with similar opinions respecting the connexion between fever and inflammation, (Prax. Med. Lib. xvii. et S. 19); we can hardly fail to subscribe to the justice of the Professor's remark, that there is a medical, as well as a moral cycle, or, to recollect the prediction of the admired Poet of the Augustan age,

> "Alter erit tum Tiphys, et altera quæ vehat argo
> Delectos Heroas; erunt altera Bella;
> Atque iterum ad Trojam magnus mittitur Achilles."

The doctrines of the Chrysippi, of modern times, who condemned bleeding and purging, have again passed away; and we have returned to the depletory method of treatment, with, I trust, more rational expectations of success, and a conviction of its utility, founded on experience. Owing to the extensive opportunities, and the more correct and philosophical views of recent observers, elucidated by pathological research, the lancet has resumed its rank in the treatment of fever; but its use is now better understood than formerly; it is considered as an useful, not as an universal remedy; and hence, we may hope that it will maintain its importance, while its powers are soberly appreciated, and due allowance is made for difference of habit, seasons, climate, and other modifying influences. In proportion, however, as it is powerful, it is a remedy of which, of all others, it is the most difficult to attain and preserve a standard value; and there is danger that the young and the sanguine—they in whom success has inspired confidence, because of their unacquaintance with the more dangerous forms of disease, should, by the incautious and indiscriminative application of a valuable resource, contribute to bring it again into disrepute:—"Sunt certi denique fines, quos ultra, citraque, nequeat consistere rectum."

On reviewing the history and revolutions of medicine then, there is, doubtless, much room for humiliation and regret. If we feel mortified in contemplating how often fancied improvements have yielded to others equally fanciful; if the errors of our fore-fathers now appear so glaringly absurd, we should remember, that they were not less satisfied of the soundness of their conclusions, than we are of that of our own. Yet, with a becoming degree of modesty, there is, also, much reason for congratulation, when we consider the advances of medical, as well as of the other branches of science, of late years; and that with the classical taste and advantages of antient, are combined the improvements of modern times.

The human intellect, in its march, has passed through many mazes and intricacies which it is in no danger of retracing, and which it has learned, in future, to avoid; and rejecting those paths which lead through abstract and fruitless speculations, to barriers which it cannot, and which true philosophy teaches that it ought not, to attempt to pass; it is content to follow the footsteps of Nature, and to confine its attention to patient observation, and cautious induction.

To a more just and extensive pathology, we join more correct and simple therapeutic views, and instead of abstract speculations concerning the principle of vitality, and proximate causes, we are becoming more and more aware of the advantages of judging according to the plan recommended by M. Corvisart: "De sedibus et causis morborum, per signa diagnostica investigatis, et per anatomen confirmatis."

We may, therefore, safely conclude in the words of Mr. Locke, and if they were applicable in his days, they must be much more so in our own: "Though we cannot say there are fewer improbable and erroneous opinions in the world than there were, yet this is certain, there are fewer people who actually assent to them, and mistake them for truths, than is imagined."

*September* 26, 1817.

